# Use and impact of the ANA Code: a scoping review

**DOI:** 10.1177/09697330241230522

**Published:** 2024-02-07

**Authors:** Olivia Numminen, Hanna Kallio, Helena Leino-Kilpi, Liz Stokes, Martha Turner, Mari Kangasniemi

**Affiliations:** 60654 University of Turku; 8277 American Nurses Association; University of Minnesota; University of Turku

**Keywords:** American Nurses Association, codes of ethics, impact, nursing, professional code, scoping review

## Abstract

Adherence to professional ethics in nursing is fundamental for high-quality ethical care. However, analysis of the use and impact of nurses’ codes of ethics as a part of professional ethics is limited. To fill this gap in knowledge, the aim of our review was to describe the use and impact of the Code of Ethics for Nurses with Interpretive Statements published by the American Nurses Association as an example of one of the earliest and most extensive codes of ethics for nurses with their interpretative statements and constituting a strong basis for the International Council of Nurses’ Code of Ethics for Nurses. We based our review on previous literature using a scoping review method. We included both non-scientific and scientific publications to provide an analysis of codes of ethics which can be utilized in development and revision of other nurses’ codes of ethics. In the searches, we used CINAHL and PubMed databases limiting publications to texts with a connection to the Code of Ethics for Nurses published from January 2001 to November 2022 and written in English. Searches yielded 1739 references, from which 785 non-scientific and 71 scientific publications were included for analysis of the data. Although non-scientific and scientific publications addressed different number of categories, the results indicated that in the both groups the use and impact focused on professional ethics, nursing practice, and work environment and less on education, research, or social health issues. Nurses’ ethical standards were not addressed in non-scientific publications, and clinical issues and leadership were not in focus in scientific publications. To increase evidence-based knowledge of the impact of codes of ethics additional research is needed. Good scientific conduct was followed.

## Introduction

Professional codes of ethics guide nurses’ practice. They describe the values and principles of the profession, which aim to support nurses’ ethical decision making and express the profession’s obligations to society. The International Council of Nurses’ Code of Ethics unifies the profession globally,^1^ while national codes respond to local nursing needs. Codes of ethics provide a foundation for nurses’ ethical competence.^2,3^ Throughout nursing’s history codes of ethics have been revised periodically due to changes in practice, technology, emerging concerns, and societal developments.^1^ Thus, professional codes of ethics are a living document, designed to guide nurses now and in the future.^4^

A profession, like nursing, is characterized by its willingness to comply with ethical and professional standards as they are defined in the profession’s codes of ethics. They also support the development of the professional group identity.^5,6^ Nurses’ codes of ethics have both external and internal functions. External functions describe the profession’s position in society, whereas internal functions describe a normative set of rules.^7,8^ As an external function, nurses’ codes of ethics express the values of the profession, the codes’ importance in the recognition of patients’ rights, and how the profession addresses ethical obligations to society.^7,9^ Thus, the professional codes of ethics serve as the social contract between the public and the nursing profession.^7^ As an internal function, codes of ethics describe standards of ethical practice, moral obligations, and acceptable behaviors.^10,11^ Codes of ethics are a form of professional self-regulation to which professionals as individuals and as a group commit themselves voluntarily.^8^

## Background

This study focuses on Code of Ethics for Nurses with Interpretive Statements published by the American Nurses Association (hereafter the Code).^2^ American Nurses Association (ANA) is a national nursing organization representing the interests of more than five million registered nurses. ANA advances the profession fostering high standards of nursing practice, promoting a safe and ethical work environment, bolstering the health and wellness of nurses, and advocating health care issues that affect nurses and the public (https://www.nursingworld.org). Identifying the use of the Code in nursing publications may reveal new applications of the Code useful for revision and development of codes of ethics.

### History of the code of ethics for nurses with interpretive statements

In 1950, ANA formally adopted a Code for Professional Nurses written to establish principles of conduct for the profession and outlined the nurse–patient relationship, nurse–physician relationship, and nurse–profession relationship. This Code contained 17 declarative provisions including topics such as the responsibility to conserve life, patient abandonment, respect for patient’s religious beliefs, confidentiality, and inter-professionalism. In responses to changes in society, changes in nursing education and practice, and advances in technology and science, two versions of the Code were published in the 1960s, which shifted the focus from conservation of life to deeper obligations respecting human dignity and safeguarding patient’s rights to privacy. An increasing awareness of the nature and determinants of global health contributed to the succeeding iterations (1976 and 1985). Interpretive statements were added to each of the provisions and gave greater depth and a broader duty to contribute to the professional body of knowledge. Continuing education and competence with an increased focus on inter-professionalism were added. By the early 2000s, the concept of compassion was emphasized and a renewed commitment to patients, families, and communities was included. The duty to self, which was dropped out of the Code after the 1960s, was also renewed focusing on nurses’ moral duties of self-respect, preservation of wholeness of character, and sound ethical decision making.

In the latest revision of the Code in 2015, there was an inclusive intent to provide a guide for all nurses, in all roles, and in all settings. The significant change in the 2015 revision process was the use of technology that enabled collaborative revision across the country without in-person meetings. Throughout, there were several new elements, for instance, expanded content, and terminology to respond to changes in care delivery, services, research, and society. The 2015 Code consists of nine provisions (Table 1) and includes interpretive statements as a resource to use and apply the Code in clinical practice, research, decision making, education, and leadership.^2^

**Table 1.** Use of the Code’s provisions in publications^a^.

	Non-scientific (*n* = 785)		Scientific (*n* = 71)	
Provision no.	n	%	*n*	%
#1- The nurse practices with compassion and respect for the inherent dignity, worth, and unique attributes of every person.	184	18	38	16
#2- The nurse’s primary commitment is to the patient, whether an individual, family, group, community, or population.	118	12	29	13
#3- The nurse promotes, advocates for, and protects the rights, health, and safety of the patient.	177	18	28	12
#4- The nurse has authority, accountability, and responsibility for nursing practice; makes decisions; and takes action consistent with the obligation to promote health and to provide optimal care.	86	9	23	11
#5- The nurse owes the same duties to self as to others, including the responsibility to promote health and safety, preserve wholeness of character and integrity, maintain competence, and continue personal and professional growth.	121	12	19	9
#6- The nurse, through individual and collective effort, establishes, maintains, and improves the ethical environment of the work setting and conditions of employment that are conducive to safe, quality health care.	96	10	23	11
#7- The nurse, in all roles and settings, advances the profession through research and scholarly inquiry, professional standards development, and the generation of both nursing and health policy.	64	6	21	10
#8- The nurse collaborates with other health professionals and the public to protect human rights, promote health diplomacy, and reduce health disparities.	75	8	19	9
#9- The profession of nursing, collectively through its professional organizations, must articulate nursing values, maintain the integrity of the profession, and integrate principles of social justice into nursing and health policy.	76	8	19	9
***In total***	**997**	**100**	**219**	**100**

^a^Several provisions in one publication.

### Previous research of the use and the impact of nurses’ professional codes

Previous research on nurses’ professional codes of ethics has mainly focused on nurses’ awareness and use of codes.^12^ In clinical practice, nurses’ adherence to the codes seems to be high,^13^ although several barriers to following the codes have been identified^14^ including individual attributes and organizational factors.^15^ Also, international comparison of the use and understanding of the content of nurses’ codes,^16^ interdisciplinary reflection, and theoretical analysis of the functions of the codes has been carried out.^17–19^ Additionally, the impact of professional codes of ethics has been investigated in several other fields, for example, in mobile journalism,^20^ biology,^21^ and eco-system survival.^22^

In health care, the impact of professional codes has been considered when identifying how they support nursing practice^23^ and the status of the nursing profession in society.^4,24,25^ There are also examples of the impact of codes of ethics identified in tele-health practices,^26^ midwifery,^27^ facilitation of the use of technology,^28^ and in collegial relationships.^29^ The impact has also been considered in relation to nursing management, use of resources and quality of care,^30^ ethical leadership,^31^ and ethical international nurse recruitment.^32^ In nursing education, codes of ethics are used especially for teaching and learning professionalism, ethical practice, professional values, and learning ethical action.^11,18^ The importance of codes of ethics in clinical practice and work life is emphasized in the literature.^14,25^

The use and impact of codes of ethics can be considered directly and indirectly. Directly, how they unify and support nurses’ practice, research, and administration. Indirectly, as the description and reflection of nursing practice. Impact can be seen as multidimensional, including the use in different contexts, and also as the outcomes and consequences of the use. Outcomes can be identified based on scientific research (e.g., Reference 33) and in narratives among professionals and professions.^34^ Indirect impact refers to how the codes have been used as a part or a tool of research and how often they have been cited, recognized, and considered.

## Aim

The aim of this review was to determine the use and impact of the American Nurses Association Code of Ethics for Nurses with Interpretive Statements. The purpose of the review was to increase understanding of the use and impact of the Code to provide evidence for further development and revision of the Code and other national and international codes of ethics in response to new challenges in nursing practice, research, policy, and education.

The research question was as follows:

— How has the use and impact of the Code been described in nursing publications?

## Method

**Figure 1.**
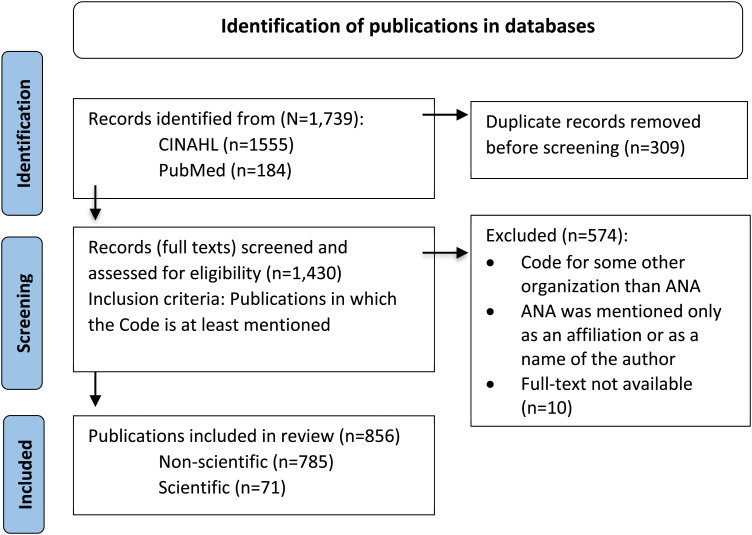
Flow chart (reported according to Page et al.^36^).

We used a scoping review based on the methodological framework by Arksey and O’Malley’s^35^ to explore literature referring to or discussing the Code (Figure 1) following five stages of the method: identifying research questions and relevant papers, selecting the papers, charting the data and collating, summarizing, and reporting the results. The review follows the Preferred Reporting Items for Systematic Reviews and Meta-Analyses (PRISMA) extension for Scoping Reviews.^37^

### Identification of research questions and relevant publications

We identified the research question based on preliminary literature searches to guide the search strategy^35^ focusing our research question to identify non-scientific and scientific publications available in electronic databases. No previous reviews were found. Next, we carried out electronic searches using CINAHL and PubMed databases covering the literature in nursing and related fields. We used Boolean operator to formulate our search phrase as follows: (ANA OR “American nurse* association”) AND (”code* of ethics” OR “ethical code*”) limiting our searches to publications published between January 2001 and November 2022 in the English language. The purpose was to analyze the use and impact of the Code over a time period covering two revisions, in 2001 and 2015, of the Code due to changes in nursing practice, advances in technology, societal changes, and expansion of nursing practice into advanced practice roles, research, education, health policy, and administration during these years.^2^

### Selecting publications

We selected publications based on full texts and using inclusion and exclusion criteria. We included publications that mentioned the Code or a section of it anywhere in the text. We excluded publications representing codes of ethics of an organization other than ANA and if the ANA was mentioned only as an affiliation or as a name of an author. Our searches yielded 1739 publications and after removing duplicates (*n* = 309), we had 1430 publications in total. Four researchers (ON, HK, HL-K, MK*)* read full texts of all publications. Based on the inclusion and exclusion criteria, we excluded 574 publications, leaving 856 publications for our data. We divided the selected publications into two categories. The first category was non-scientific publications, comprising various types of articles published in professional nursing journals, and the second category was scientific publications, comprising empirical studies and theoretical discussion papers published in peer-reviewed scientific journals yielding 785 non-scientific and 71 scientific articles (Figure 1).

### Charting the data and data analysis

From non-scientific publication category we extracted author(s) name, year, country, and title of each publication and from scientific publication category author(s) name, year, country, title, and also method. We analyzed the data using both deductive and inductive reasoning.^38^ The Code was used as a deductive framework.^38^ We extracted all phrases referring to the entire Code or an individual provision(s) and calculated the frequency of these phrases in the publications (Table 1). Thereafter, we analyzed data inductively^38^ identifying the main topic of nursing from each publication and based on their similarities and differences, grouped them first in the sub-categories and again to main categories. We named all categories inductively based on their content. As a result, we had thirteen sub-categories and seven main categories describing nurses’ professional activities. The frequency of different professional activities within the categories was then calculated (Table 2). In the scientific publications, the analysis was continued analyzing also the main results and methods more in detail (Table 3).

**Table 2.** Categorization of non-scientific (*n* = 785) and scientific (*n* = 71) publications.

Nurses’ professional activity category	Non-scientific (*n* = 785)		Scientific (*n* = 71)	
	*n*	%	*n*	%
Professional ethics and good care	**316**	**41**	**34**	**48**
Codes of ethics in nursing	98	13	5	7
Professional ethics	116	15	15	21
Ethical principles in patient care	102	13	14	20
Nursing practice	**212**	**27**	**17**	**24**
Nursing fields	47	6	4	5
Topics in nursing	111	14	9	13
Clinical issues	44	6	—	
Ethics and law	10	1	2	3
Standards of practice	—	—	2	3
Work environment and nurses’ working life	**133**	**17**	**13**	**18**
Working life	56	7	2	3
Working community and collaboration	31	4	7	10
Nurse’s role and identity	17	2	4	5
Nurse’s rights	13	2	—	—
Workforce issues	16	2	—	—
Association activities and membership	**70**	**9**	—	—
Leadership	**23**	**3**	—	—
Education	**17**	**2**	**6**	**9**
Research	**3**	**1**	**1**	**1**
Other	**11**	**1**	—	—

**Table 3.** Main results of scientific publications (*n* = 71).

Category	Author/s, year	Method	Main results
Professional ethics and good care (*n* = 34)			
• The Code (*n* = 5)			
Development	Philpin and Keepnews (2014)^39^	Historical	ANA leaders’ and nurses’ leadership, commitment, and contribution accomplished a code of ethics, stating nurses’ ethical obligations, standards, and commitment.
Purposes and functions	Schmidt et al. (2017)^40^	Theoretical	The Code should be a living document.
	Epstein and Turner (2015)^41^		The Code’s functions impact nursing education, practice, research, and policy.
	Dahnke (2009)^6^		An understanding of the Code supports nurses’ ethical judgment increasing autonomy.
Critique	Vogelstein (2016)^42^	Critical analysis	Professional health care organizations should take common and justified stances in professional ethics.
• Professional ethics (*n* = 15)			
Nurses’ duties	McNeill et al. (2020)^43^	The Nash Duty to Care Scale, convenience sampling/snowball technique, nurses (*n* = 289), statistical analysis	Duty to care in disasters was driven by the Code.
Morality and virtues	Burnell (2011)^44^	Interview, convenience sampling, out-patient clinic clients (*n* = 26), interpretive analysis	Nurses should respond with compassion in caring.
	Sullivan (2012)^45^	Interview, purposive sampling, nurses (*n* = 7) as key informants and clinical nurse educators (*n* = 14) as informants, Leininger’s research enablers analysis	Nurses’ ethical commitment to the patient was (1) striving to enact commitment, (2) developing a voice to enact commitment, and (3) institutional practices as facilitators or barriers to enact commitment.
Values	Schank and Weis (2001)^46^	Nurses Professional Values Scale (NPVS), convenience sampling, nurses (*n* = 22) and nursing students (*n* = 29), statistical analysis	Professional values dealt with value measurement using Nurses Professional Values Scales (NPVS), based on the Code.
	Weis and Schank (2009)^47^	Psychometric evaluation on NPVS random sampling, nursing students (*n* = 404), graduates (*n* = 80), and nurses (*n* = 298), statistical analysis	Psychometric properties of the NPVS-R and NPVS-3 proved valid. Nurses and students had a good knowledge of values, higher scores connected to ethics education. Nurses’ and students’ significant differences were in the importance of values. Important values concerned patient care rather than professional associations, research, and policy development. Nurses’ knowledge exceeded that of students. Personal demographics, experience, education, geographic location, and cultural background influenced values. The integration of values into nursing education was essential. Value internalization requires staff educators.
	LeDuc and Kotzer (2009)^48^	NPVS, convenience sampling, nursing students (*n* = 97), graduates (*n* = 46), and nurses (*n* = 84), statistical analysis	
	Alfred et al. (2013)^49^	NPVS-Revised, convenience sampling, nursing students (*n* = 262), statistical analysis	
	Feller (2014)^50^	NPVS-R, purposive sampling, nursing students (*n* = 185) and nursing science students (*n* = 37), statistical analysis	
	Posluzny and Hawley (2017)^51^	NPVS-R, convenience sampling, nursing students (*n* = 136), statistical analysis	
	Weis and Schank (2017)^52^	NPVS-3, random sampling, nurses and nursing students (*n* = 1139), statistical analysis	
	Feller et al. (2019)^53^	Secondary analysis, NPVS-R, convenience sampling, senior nursing students (*n* = 417), statistical analysis	
	Monroe (2019)^54^	NPVS-R, convenience sampling, nurses (*n* = 2439), statistical analysis	
	Knecht et al. (2020)^55^	NPVS-R, purposive sampling, nursing students (*n* = 119), statistical analysis	
Decision making	Wueste (2005)^56^	Theoretical	The Code was a resource providing viewpoints, for example, use of ethical theories, cultivating virtues, strengthening nurses’ moral position within health care, and supporting evidenced-based decision making.
	Bold (2012)^57^	Self-developed questionnaire, convenience sampling, nurses (*n* = 26), statistical analysis	
• Ethical principles in patient care (*n* = 14)			
Advocacy and accountability	Kalaitzidis and Jewell (2020)^58^	Theoretical	Speaking on behalf of the patient seems the commonly accepted definition for advocacy.
	Berlandi (2002)^59^		Accountability and responsibility are strengths in perioperative nurses’ decision making.
Human dignity and respect	Jacelon et al. (2004)^60^	Concept analysis	Human dignity obligates nurses to treat patients with dignity regardless of illness or behavior. Students’ knowledge of human dignity and practice validated the importance of using the Code as a foundation for ethics education in advanced practice nursing. According to the Code, nurses should base their V.I.P. patients’ care on patients’ health needs and respect.
	Green (2011)^61^	Theoretical	
	Kalb and O’Conner-Von (2007)^62^	Interview, self-developed questionnaire, convenience sampling, nursing students (*n* = 63), content analysis	
	Perrone (2020)^63^	Theoretical	
	Beckemeier and Butterfield (2005)^64^		
Social justice and social ethics/policy	Valderama-Wallace (2017)^65^	Discourse analysis	The Code, Social Policy Statement, and Scope and Standards of Practice revealed inconsistencies in conceptualization of social justice offering an inadequate framework for nurses. Potential conflicts between a patient-focused approach and nurses’ social and political responsibilities need critical analysis in terms of nurses using professional power in education, practice, and policy development.
	Garcia (2021)^66^	Theoretical	The Code outlines nurses’ responsibilities based on ethics, human rights, and social justice.
	Waite and Nardi (2021)^67^		The Code denies antiblack racism requiring nurses’ antiracist actions, the respect for dignity, and human rights.
	Fowler (2016)^4^		The Code provides a moral demand for nursing participation in national and global health policy. Promoting collaboration with other health professions helps nurses to realize the ends of social ethics.
Patient vulnerability	Gathron (2019)^68^	Concept analysis	The Code helps to advocate in risk of vulnerability of seriously sick.
	Fitzgerald (2021)^69^	Theoretical	
Patients’ rights.	Murray (2003)^70^	Focus group (*n* = 7) interview, purposive/snowball sampling, women (*n* = 14) and men (*n* = 4), constant comparative analysis (Glaser & Strauss, 1967)	Emerged patients’ rights substantiated the Code’s value as a framework for practice.
Nursing practice (*n* = 17)			
• Nursing fields (*n* = 4)			
Perioperative	Seifert (2008)^71^	Theoretical	Perioperative nurses must know the Code to provide quality care and strengthen their moral position in health care.
Pediatric/psychiatric	Regan (2010)^72^	Theoretical	The Code guided nurses in ethical dilemmas.
Intensive care	Parsons and Walters (2019)^33^	Theoretical	The Code reinforced nurses’ psychosocial care entailing treating patients with dignity and respect.
Public health	Ivanov and Oden (2013)^73^	Theoretical	The Code guides public health nurses to avoid restriction of individual rights as a serious deviation.
• Nursing topics (*n* = 9)			
Disasters and pandemics	Esposito and Sollazzo (2018)^74^	Interview, purposive sampling, nurses (*n* = 6), sociologist (*n* = 1), doctor of Nursing Practice (*n* = 1) and drivers (*n* = 2), interpretive analysis	The Ethical Codes of Conduct and Human Rights Obligations guided nurses working in disasters. The Code did not offer guidance how nurses handle their guilt for the lack of support for isolated COVID-19 patients.
	Dellasega and Kanaskie (2021)^75^	Focus group (*n* = 6) interview, convenience sampling, nurses (*n* = 23), thematic analysis	
	Carnevale et al. (2009)^76^	Narrative review	In cross-linguistic nursing, the patient’s self-determination, privacy and confidentiality, responsibility for one’s own competence, and moral action meeting patients’ needs referred to provisions #1–#6.
End-of-life care and assisted suicide	DeWolf et al. (2013)^77^	Self-developed questionnaire, purposive sampling, nurses (*n* = 13), statistical analysis	Nurses’ values on patient-directed-dying and its consistency with the Code revealed that nurses understanding the Code well supported PDD (patient-directed-dying), whereas nurses whose understanding was deficient were unsure of PDD. Initiating PDD, advanced practice nurses reported an increased willingness to discuss and prescribe.
	Jannette et al. (2013)^78^	Self-developed questionnaire, convenience sampling, Advanced Practice Nurses (*n* = 16), statistical analysis	According to the Code, it is ethical to deactivate a life-sustaining device at the patient’s request due to the patient’s self-determination.
	Gura (2015)^79^	Theoretical	ANA should have a convincing justification for opposing the nurse’s participation in assisted suicide.
	Vogelstein (2019)^80^	Critical analysis	
Genomics and bioethics	Dugas (2005)^81^	Theoretical	In genetics nursing the Code emphasized evidence-based knowledge in informed consent, patient privacy, confidentiality, self-determination, discrimination, and values. Nurses should collaborate with policymakers, clinicians, researchers, and educators in identifying ethical genetic issues and demonstrate leadership in inter-professional initiatives in establishing national/international guidelines.
	Tluczek et al. (2019)^82^		
• Ethics and law (*n* = 2)			
Nurses’ role in end-of life care	Clymin et al. (2012)^83^	Interview, self-developed questionnaire, convenience sampling, nurses (*n* = 582), content analysis	Nurses’ lacked knowledge and education of Washington State’s Death with Dignity Act. Nurses’ role in implementation of this act required clarification in relation to the Code. The Code and ANA position statements on assisted suicide and end-of-life care guide nurses.
	Jablonski et al. (2012)^84^	Self-developed questionnaire, convenience sampling, nurses (*n* = 582), statistical analysis	
• Standards of practice (*n* = 2)			
Professional self-regulation and breaches of professional standards	Olson and Stokes (2016)^85^	Theoretical	The Code is a tool for profession’s self-regulation and a resource for nurses’ ethical decision making. Nursing students needed the Code to define the ethical obligations and duties in observing nurses’ breaching of standards of practice.
	Wentworth et al. (2020)^34^	Interview, purposive sampling, nursing students (*n* = 10), phenomenological data analysis/Colaizzi method	
Work environment and nurses’ working life (*n* = 13)			
• Working life (*n* = 2)			
Workplace violence and professional boundaries	Copeland (2021)^86^	Discourse analysis	In workplace violence, the Code defines the ethical standards for the profession. Nurses must take care of their own well-being and respect their boundaries in fulfilling their commitments to others.
	Nelson and Rushton (2021)^87^	Theoretical	
• Working community and collaboration (*n* = 7)			
Social networking	Simpson (2005)^88^	Theoretical	Nurses must take responsibility in using social networking. Nurse leaders’ obligation is to inform nurses of the use of electronic health records. Electronic health systems may erode the respect for humanity. Provisions #1, #2, and #6 provide guidance in electronic mediation in health care. The Code emphasizes nurses’ duty to maintain vigilance in use of social media. Untoward use of social media is a breach of the Code.
	McBride et al. (2018)^89^		
	Rentmeester (2018)^90^		
	Catlin (2013)^91^		
	Westerick (2016)^92^		
	Daigle (2020)^93^		
	Lancaster et al. (2022)^94^	Videos (*n* = 52), purposive sampling, content analysis	
• Nurse’s role and identity (*n* = 4)			
Nurse/researcher role	Judkins-Cohn et al. (2014)^95^	Theoretical	The Code can support nurses in clinical and research settings, the Code helping to improve understanding the differences between the roles.
Informed consent	Cook (2014)^96^	Theoretical	Using the Code and evidence-based practice, nurses’ action in the informed consent process becomes better justified.
Nurse identity	Kern (2006)^97^	Phenomenological interview, purposive/convenience sampling, nurses (*n* = 8), analysis/van Maanen method	Most nurses felt they were still nurses, even though they were not any more in practice, the Code as the ethical model and Carper’s (1978) four Patterns of Knowing as the epistemic model in examining development of nurse identity.
	Reyes-Villacomeza (2009)^98^	Interview, purposive, snowball/theoretical sampling, nurse–physicians (*n* = 12), constant comparative analysis	Development of foreign-educated physicians’ nursing identity was operationalized by utilizing three statements of the Code.
Education (*n* = 6)			
Educational content and methods	Wilk and Bowllan (2011)^99^	Quality improvement project report	The Code is an essential content and should be taught from different perspectives also acting as a guide for students. The Code, ethics terminology, and institutional integrity policies are important in teaching ethics. Nursing students had ability to apply the Code, simulation, vignettes, and the Code as teaching material. Students lacked familiarity with ethics consultation and ethical principles based on the Code in comparison with students participating in ethics consultation and students who were taught using traditional methods.
	Laabs (2015)^100^	Delphi technique with three round online surveys, qualitative and quantitative data, purposive sampling, ethics experts (*n* = 29/15/12), qualitative and statistical analyses	
	Beck (2018)^101^	Self-developed Nurse Clinical Ethics Survey (NCES), convenience sampling, nursing students (*n* = 322), statistical analysis	
	Greenawalt et al. (2017)^102^	Self-developed questionnaire, convenience sampling, nursing students (*n* = 93), statistical analysis	
Student misbehavior	Donnelly et al. (2017)^103^	Quasi-experimental study with control and experimental groups, the pre-test/post-test design intervention, random sampling, nursing students (*n* = 167/154/145), statistical analysis	Higher level education and paying for education had a significant positive effect on ethical behavior regardless of demographics.
Student payment	Copeland (2020)^104^	Theoretical	Paying students for their clinical placements was a challenge, disadvantages seeming greater than the advantages added with unknown long-term consequences, the Code providing guidance.
Research (*n* = 1)			
	Smirnoff et al. (2007)^105^	Self-developed questionnaire, convenience sampling, nurses (*n* = 470), statistical analysis	Despite emphasis on the Code, nurses’ attitudes toward nursing research were discordant with their involvement with research activities.

## Results

Based on our literature searches, we selected 785 non-scientific and 71 scientific publications for data. Non-scientific articles fell into seven and scientific articles into five main categories concerning nurses’ professional activities described in the analyzed publications the context of the Code, in which emphasis was in professional ethics and good care, and nursing practice (Table 2). The most frequently and the least referred provisions were in the same line emphasizing immediate nurse–patient relationship, whereas the nurse’s larger societal and researcher roles were less in focus (Table 1).

### Characteristics of the publications

**Figure 2.**
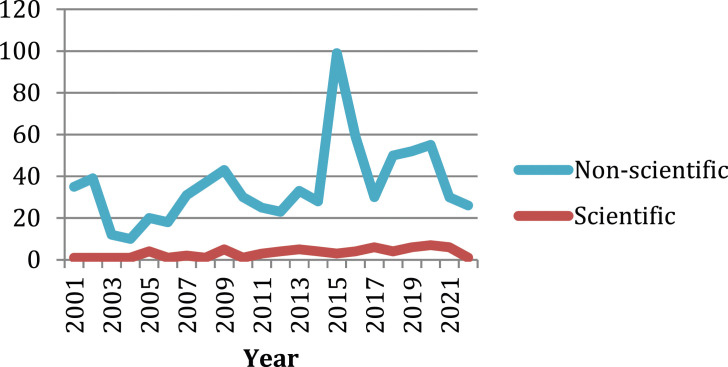
Non-scientific (*n* = 785) and scientific (*n* = 71) publications with the Code according to the year of publishing.

***Non-scientific*** *publications* (*n* = 785) were mostly professional articles (*n* = 487; 82%) (Table 4), where the Code was referred in relation to various topics of nursing. Often the publications were columns from professional nurses’ associations (*n* = 120; 15%) and information bulletins (*n* = 59; 8%). The Code was reprinted in ten publications. There was a momentary increase in non-scientific publications after launching the updated Code in 2015 (Table 4, Figure 2).

**Table 4.** Characteristics of the non-scientific publications (*n* = 785).

Year of publication	*n*	%
2001–2014	384	49
2015–2022	401	51
***In total***	**785**	**100**
Type of publication		
Professional publications	**596**	**76**
Articles	487	62
Statements and position papers	35	4
Editorials	18	3
Study reports	18	3
Proposals and resolutions	11	2
Comments, commentaries, and responses	11	2
Reference reports and proposals	4	—
Closing words	3	1
Letters to the editor	2	—
Columns	2	—
Reports	2	—
Case study exercises	2	—
Guidelines	1	—
Messages from associations	**120**	**15**
Presidents’ letters	99	12
Bylaws and bylaw changes	12	2
People introductions	6	1
Information	2	—
Election policies	1	—
Information	**59**	**8**
Newsletters	33	4
Announcements	11	2
Conference news and reports	9	1
Advertisements	6	1
Reprint of the ANA Code of Ethics	**10**	**1**
***In total***	**785**	**100**

***Scientific publications*** (*n* = 71) were either theoretical (*n* = 40; 56%) discussions papers of various topics or empirical studies (*n* = 31; 44%) analyzing topics with collected data. All were published in scientific journals. Eight empirical publications were doctoral dissertations. In scientific publications the Code was in focus directly (*n* = 5; 7%), in relation to some topic of nursing (*n* = 39; 55%), or the Code was only mentioned (*n* = 27; 38%). Thirty-four publications (48%) covered the years 2001–2014 and thirty-seven (52%) the years 2015–2022. The origin of all publications was the USA, one originated from Canada, and one from Australia. The number of scientific publications did not show any marked change in the years 2001–2022 (Table 4, Figure 2).

In empirical studies registered nurses were the largest group of participants with sample sizes ranging from 6 to 2439, followed by nursing students with sample sizes ranging from 10 to 417, and one study using nurses and students (*n* = 1139). Other participant groups were ethics experts, and key or general informants and nurse–physicians referring to foreign physicians practicing as nurses in the USA.

### The code in the publications

***Non-scientific*** *publications* referred most frequently to provisions #1 and #2 concerning nurses’ duty to respect every person and responsibility to promote and advocate patients’ rights and safety. The least referred provisions were #7 related to research and development and provisions #8 and #9 concerning nurses’ responsibilities for public and profession (Table 1).

***Scientific*** *publications* referred most frequently to provisions were #1–#3 concerning nurses’ duty to respect every person, nurses’ commitment, and responsibility to promote and advocate patients’ rights and safety. The least frequently referenced provisions (#7–#9) concerned nurses’ professional development through research, health policy, integrity of the profession, and social justice (Table 1).

### Nurses' professional activity categories in the publications

***Non-scientific*** *publications* addressed topics in seven large nurses’ activity categories (Table 2). Often the focus was on *Professional ethics and good care* (*n* = 316; 41%) including codes of ethics in nursing, professional ethics and ethical principles in patient care and addressing such issues as the Code itself, professionalism, nurses’ duties, and advocacy. Another large activity category was *Nursing practice* (*n* = 212; 27%) where the Code was related to different fields, topics, clinical issues, ethics and law, and standards of practice addressing disasters and pandemics, racism, and quality care in many nursing contexts. The third large activity category was *Work environment and nurses’ working life* (*n* = 133; 17%) including issues in working life, working community and collaboration, nurses’ role and identity, nurses’ rights, and workforce concerns. These concerns dealt with workplace violence collaboration and staffing. The fourth large activity category addressed *Association activities and membership* issues (*n* = 70; 9%). Less frequently addressed activities were *Leadership* (*n* = 23; 3%), *Education* (*n* = 17; 2%), and *Research* (*n* = 3; 1%). Detailed results are reported in Table 2 and Appendix 2.

***Scientific*** *publications’* (*n* = 71) topics fell into five professional activity categories (Table 2). The most frequently referred activity category was *Professional ethics and good care* (*n* = 34; 48%). Results showed that the Code should be used continually, and that it has impact on nursing education, practice, research, and policy-making increasing nurses’ autonomy in ethical judgement. The Code defined nurses’ duties, virtues, values, and ethical principles and supported ethical decision making and deliberation. Social justice, human dignity, advocacy, vulnerability, and patients’ rights were seen as important.^4,6,39–46,50–52,54–70^ The second most frequently referred activity category was *Nursing practice* (*n* = 17;24%). The results revealed that the Code guides and reinforces nurses in different nursing fields and contexts such as disasters, racism, genomics, end-of-life issues, law and ethics, and professional standards, but nurses also needed further education and guidance in many of related issues.^33,34,71–85^ The third activity category dealt with *Work environment and nurses working life* (*n* = 13; 18%) dealing with workplace violence, nurses’ self-care, social networking, and nurses’ role and identity. In all these, the Code was setting the standards of behavior and professional boundaries. The Code was also used as a framework in discussing nurse identity.^86–98^ The fourth activity category of scientific publications concerning *Education* (*n* = 6; 9%) showed that teaching of the Code from several perspectives was essential.^99–104^ The least referenced activity category was *Research* (*n* = 1; 1%), revealing that nurses’ participation in research activities is limited.^105^ Detailed results are reported in Table 3.

## Discussion

This scoping review analyzed the use and impact of nurses’ codes of ethics focusing on the American Nurses’ Association Code of Ethics (the Code) as an example.^2^ The Code has been updated periodically due to changes in practice, technology, emerging concerns, and societal development, both nationally and globally.^2^ The literature comprised non-scientific and scientific publications covering the years 2001–2022 and addressing a heterogenous group of nursing fields, topics, and nursing contexts. Use of non-scientific and scientific literature produced two kinds of knowledge of the Code’s impact: empirical research and theoretical analyses are needed to support the codes’ evidence-based development and use, while not underestimating the impact of non-scientific literature as the reflection of nurses’ daily practice concerns in revising nurses’ codes of ethics.^106,107^

In the publications, the main focus was on professional ethics and good care, suggesting a conventional approach in the use and impact of the codes. Interest in direct patient care and professional ethical issues emphasized care on micro- and meso-level nursing.^4^ This finding conforms with earlier studies of the primacy of nurses’ work orientation concentrating on the nurse–patient relationship in clinical settings.^108^ Less attention was paid to larger social or leadership issues which would point to macro-level nursing care.^4,109,110^ However, as the largest group of health care professionals globally,^4^ we need to critically analyze factors that promote or prevent nurses’ fulfilling their societal mandate, and consequently its impact. The mandate is clearly expressed in most nurses’ codes of ethics.^1,2,4^ Nurses’ codes of ethics have both internal and external functions. However, there is little knowledge of how the codes are reflected outside the profession and in collective decision making. This knowledge is important to assess the impact of the codes of ethics on patient care from a wider perspective. Particular attention should focus on nurses who, based on their professional position, are expected to look at nursing from larger perspectives. Professional values were studied rather systematically using versions of Nurses Professional Value Scale^46^ comprising factors of caring, activism, trust, professionalism, and justice^46^ based on the Code. These value studies are a good example of systematic research in nursing ethics. Many ethical areas in nursing, including codes of ethics, beg for developing instruments and interventions and investing in multifaceted research designs.^111^ In issues concerning nursing practice, work environment, and nurses’ working life, the impact of the Code was mainly seen as a guide and support for deliberate problematic issues.^6,46,63^ Education and research were less in focus. Because publications did not directly focus on teaching codes of ethics, the importance of education may be underestimated in the reviewed literature. Several empirical studies used nursing students as participants, in which importance of teaching codes of ethics was brought up as an essential element of ethics education, as it has been for a long time (e.g., References 24, 112, and 113). As to research, the findings are in line with many studies indicating nurses’ limited use of research in guiding their practice or participating in research activities.^1,2,114^

Critical discussion of the Code was quite modest (e.g., Reference 42). The codes can be questioned if and how they guide the nursing profession. They should be practiced, not only talked about.^11,115^ Research has shown that many barriers, such as personal and organizational factors, prevent their use.^12,14,109^ Furthermore, codes of ethics are known to have several common limitations, such as weaknesses in philosophical foundations, their normative and prescriptive nature, or their minimal impact on moral behavior. The first refers to argumentation from authority and arbitrary choice of values, the second to morality being more than a set of rules, and the last suggesting that the codes do not necessarily impact moral behavior.^116,117^ Even the claim that codes can be replaced with laws and other documents regulating health professions has been suggested.^118^ However, as a caring profession, codes of ethics are seen as fundamental for nursing, supporting the good of patients. This fundamental meaning is still important and relevant.^24^ As a normative set of rules, codes of ethics define what is regarded as right and good in nursing. These definitions need revision from time to time. For example, today’s serious global challenges, such as the COVID-19 pandemic and environmental changes, have huge implications for the nursing profession in identifying new ethical goals. These goals clearly involve a need for participating in society to secure healthy living conditions for future generations on the earth^119^ by environmentally responsible ways of practicing nursing.^120^

Furthermore, global immigration continues to bring up new cultural challenges requiring complex responses from nurses in all settings not forgetting the internationalization of nursing workforce itself. It raises the question of the need of developing global codes of ethics for nurses in which the profession’s basic ethical principles and values are expressed and appreciated in every country and every situation at all levels of nursing care including care cultures in countries in which ICN code of ethics is not in use. In this, the new Code of Ethics of International Council of Nurses^1,2^ and all national codes including the ANA Code provide a good starting point for collaboration utilizing both evidence-based scientific knowledge as well as knowledge from nursing practice published in professional journals in analyzing the use and impact of the codes of ethics and in developing global approach to the codes for the best of patients and nurses themselves. However, due to differences between countries and their care cultures we should not undermine the value of national codes. They bring ethical issues in professional practice visible, respond to societal needs concerning ethical issues in health care, and reflect the basic function of the profession as well as strengthen the profession’s justification to provide nursing care.^121,122^

## Limitations and future research

There are some minor limitations in this scoping review concerning the focus, literature searches, and data analysis. One limitation is focusing the review on the code of ethics of one nursing organization. However, that enabled us to examine the extent, range, and nature of publication activity around a limited topic in the codes that has not been comprehensively reviewed before.^35^ In the future, the use and impact of nurses’ codes of ethics needs more extensive research focusing also on national codes of smaller countries and their comparison. In addition, comparative research between national codes and other health care professions’ codes of ethics might reveal common perspectives for decision making in shared ethical dilemmas.

We limited our literature searches to the English language because presumably most writing of the ANA code is in English. From the viewpoint of international readership, English is also the lingua franca of science. Furthermore, our resources were limited to allow the use of translation services. The time limitation of data was based on over a time period covering two revisions, in 2001 and 2015, of the Code due to changes in nursing practice, advances in technology, societal changes, and expansion of nursing practice into advanced practice roles, research, education, health policy, and administration during these years.^2^ These limitations could be a risk that potentially relevant publications could have been missed. However, our data was large and heterogenous, consisting of both non-scientific and scientific publications covering a rather long time period. However, the scoping review method enabled us to map multiple data and present it in a summarized format.^35^

The findings of this review represent two levels of evidence, that is, professional and research-based. As a source of knowledge, these levels are differing in value.^38^ However, this division was justified to obtain knowledge both from the worlds of nursing practice and nursing science to get a comprehensive description of the use and impact of nurses’ codes of ethics for their future analysis and development. However, this approach caused some limitations. Due to a large number of non-scientific publications, only their main content was analyzed, however, demonstrating their impact and expressing nursing’s interest in informing about the codes. Perhaps more strict inclusion criteria for non-scientific articles would have allowed their deeper analysis, for example, selecting only professional articles published by ANA. Furthermore, it should be also noted that the yearly number of scientific publications was fairly scarce taking into account the time span of over two decades they covered rendering their findings rather scattered.

The focus solely on the Code unavoidably provides an American perspective to nurses’ codes of ethics. Therefore, comparative research between national codes in terms of similarities and differences in developing more uniform codes of ethics for nurses globally would be welcome. In this vein, relating to the Code of International Council of Nurses as a root of most nurses’ codes of ethics would be a natural approach to bring the Code and ICN code and all national codes of nurses even closer to each other.

The use and impact of nurses’ codes of ethics needs more extensive research focusing also on national codes of individual countries and their comparison. Comparative studies among other health care professions’ codes of ethics might reveal common perspectives for decision making in shared ethical dilemmas.^12^ Research is needed on factors influencing internalization of the codes^15^ and how the emphasis of nurse–patient relationship correlates with social justice, policy issues, and obligations (e.g., Reference 123). Methodologically, long-term research on specific code-related phenomena and analyses from various perspectives with multiple methods might provide new viewpoints and is still topical.^15,124^

## Conclusion

This study analyzed the use and impact of the Code of Ethics for Nurses with Interpretive Statements (the Code) published by the American Nurses Association on nursing, selecting it as one of the oldest and largest codes of ethics in nursing. Findings indicate the main emphasis is on the direct nurse–patient relationship, despite the Code’s guidance to get nurses involved in larger social and global issues. Education, leadership, and research were not found to be priorities. Further research of the meaning and impact of nurses’ codes of ethics is needed based on this analysis to understand the codes’ relevance to nurses. In many countries, nurses’ national codes of ethics are under development. Hopefully, this review provides new perspectives for the creation and revision of nurses’ codes of ethics and innovative ideas for further research.

## Supplemental Material

Supplemental Material - Use and impact of the ANA Code: a scoping review - seems ok - no revisions Supplemental Material for Use and impact of the ANA Code: a scoping review - seems ok - no revisions by Olivia Numminen, Hanna Kallio, Helena Leino-Kilpi, Liz Stokes, Martha Turner, and Mari Kangasniemi in Nursing Ethics.
