# Supplementary material for: Use and impact of the ANA Code: a scoping review
Source: Nurs Ethics. 2024 Feb 7;31(8):1389–412. doi: 10.1177/09697330241230522 (PMC11577685; doi:10.1177/09697330241230522)
Supplement: Supplemental Material - Use and impact of the ANA Code: a scoping review - seems ok - no revisions [file sj-pdf-1-nej-10.1177_09697330241230522.pdf]

Appendix 1. Author(s), year and aims of scientific publications (n=71).

**Author/s, year**	**Aim**
Theoretical publications (n=40)	
Berlandi 2002	To help perioperative nurses to relate the Code's Provision 4 to their practice area concerning accountability and responsibility
Jacelon *et al.* 2004	To develop a definition of dignity for older adults
Beckemeier & Butterfield 2005	To provide a critical review of the Code, Nursing's Social Policy Statement, and Nursing
Dugas 2005	To address nurses' use and application of the Code with clients with genetic dilemmas to establish professional behaviors in ethical issues in genetics.
Simpson 2005	To discuss the ethical dilemmas related to information technology in healthcare
Wueste 2005	To discuss a philosophical framework for ethical decision-making in critical care nursing
Seifert 2008	To discuss and compare the year 1985 and 2001 versions of the Code in the perioperative nursing
Carnevale *et al.* 2009	To review empirical evidence and ethical norms in cross-linguistic nursing
Dahnke 2009	To present a historical and theoretical analysis of the Code to provide for an understanding how it is to be used as an aid to moral thinking
Green 2010	To weigh and balance the ethical arguments for and against the use of "sin taxes" in mitigating preventable illnesses
Regan 2010	To discuss the use of the Code for its relevance and support for nurses experiencing ethical dilemmas.
Wilk & Bowllan 2011	To explore nursing students' perspectives on behavioral expectations that align with the Code
Catlin 2013	To consider the ethics of boundaries and nursing practice. National and international regulations are discussed using the Code as a framework
Ivanov & Oden 2013	To examine ethics from historical and practice perspectives, and to look at human rights and rights-based approach to public health nursing
Cook 2014	To examine the value of nursing's role in informed consent process
Judkins-Cohn *et al.* 2014	To describe the ethical principles of autonomy, beneficence, and justice within the nurse researcher–participant relationship
Philbin & Keepnews 2014	To explore the ANA's adoption of the Code for Professional Nurses in 1950
Epstein & Turner 2015	To discuss ethics in society, professions, and nursing and to illustrate how a professional code of ethics can guide nursing practice in all settings
Gura 2015	To educate clinicians about the legal and ethical principles that underlie withdrawal of life-sustaining therapies and to highlight the importance of proactive communication with patients and families
Fowler 2016	To discuss the impact of the Code in relation to nurses' role in patient care, social ethics and social policy
Olson & Stokes 2016	To discuss the Code as the profession's ethical standard of practice. To describe how nurse regulators use the Code to support decision-making
Vogelstein 2016	To argue that professional healthcare organizations ought not to take controversial stances on professional ethics
Westrick 2016	To discuss the promotion of professionalism in nursing students in use of electronic and social media
Schmidt *et al.* 2017	To translate nursing codes of ethics and common professional nursing values into inclusive behaviors via a code of conduct
Valderama-Wallace 2017	To examine and uncover dynamics related to power, language, and inequality within the Code, Scope and Standards of Practice, and Social Policy Statement. To examine conceptualizations of social justice
McBride *et al.* 2018	To identify and address ethical issues in use of electronic health records.
Rentmeester 2018	To argue that electronic health systems have the possibility of eroding the respect for humanity
Parsons & Walters 2019	To focus on psychosocial outcomes associated with patients, their families, and nurses who practice on ICUs
Gathron 2019	To introduce the concept of vulnerability in health care.

Tluczek *et al.* 2019	To assist nurses in interpreting the Code in genetics/genomics.
Vogelstein 2019	To evaluate ANA's argument against nurse participation in assisted suicide.
Copeland 2020	To explore some of the advantages and disadvantages if schools of nursing pay clinical agencies to host nursing students
Daigle 2020	To discuss the current challenges associated with social media of nursing students, discuss potential solutions and options and emphasize the role of nurse educator
Kalaizidis & Jewell 2020	To analyze the concept of advocacy whether there is a clear understanding of the term.
Perrone 2020	To explore the implications and ethical considerations of V.I.P. care
Copeland 2021	To critically examine ANA position statement on workplace violence through the lens of the Code.
Fitzgerald 2021	To discuss practical strategies to activate the Code which can mitigate the risks of stigmatization and vulnerability of seriously sick children
Garcia 2021	To present a formal theory analysis combined with recommendations for the use of social justice in obstetric nursing violence
Nelson & Rushton 2021	To discuss whether nurses' ought to work while ill.
Waite & Nardi 2021	To define whiteness, white supremacy, racialization, antiblack racism, white privilege, and historical trauma, and to describe intentional, persistent antiracism actions and what nurses and society need to do: to build knowledge and understanding through education, to assure health equity for individuals, populations, and communities
Quantitative publications (n=20)	
Schank & Weis 2001	To examine professional values of nursing students and practicing nurses.
Smirnoff *et al.* 2007	To collect baseline data on nurses' attitudes toward nursing research, perception of the institution as a research environment, and personal involvement in research activities.
LeDuc & Kotzer 2009	To examine the value orientation of students, new graduates, and practitioners of the fundamental values of professional nursing as presented in the Code.
Weis & Schank 2009	To examine the psychometric properties of The Nurses Professional Values Scale-Revised (NPVS-R) based on the Code
Bold 2012 *(Dissertation)*	To identify ways to enhance nurses' abilities to make ethical decisions during a disaster. The Code supports evidenced-based decisions in disasters and all nursing settings.
Jablonski *et al.* 2012	To determine nurses' level of knowledge and personal views about Washington State's (USA) Death with Dignity Act.
Alfred *et al*. 2013	To examine nursing students' professional values from two cultural perspectives.
DeWolf *et al.* 2013	To explore nurses' attitudes and values regarding patient-directed dying and perceptions of consistency or inconsistency with the Code.
Jannette *et al*. 2013	To describe the intended actions of advanced practice registered nurses (APRNs) toward initiating patient-directed dying (PDD) and prescribing a lethal dose of medication under PDD legislation.
Feller 2014 *(Dissertation)*	To determine nursing students' professional values and differences between the values based on students' program type, delivery method, and demographics.
Laabs 2015	To develop a consensus as to the essential content and methods of ethics education for advanced practice nurses.
Donnelly *et al.* 2017	To determine if participation in an ethics consultation simulation increased nursing students' knowledge of nursing ethics principles compared to students who were taught ethics in the traditional didactic format.
Greenawalt & O'Hara 2017	To evaluate undergraduate nursing students' ability to apply the Code using simulation cases.
Posluzny & Hawley 2017	To examine, what is the importance of professional values for beginning and graduating baccalaureate nursing students, and are there differences in professional values between the students.
Weis & Schank 2017	To evaluate the psychometric properties of NPVS-3
Beck 2018 *(Dissertation)*	To determine the prevalence of self-reported dishonest behaviors among baccalaureate nursing students in the clinical setting.
Feller *et al.* 2019	To carry out a secondary analysis of original data collected in three descriptive studies
Monroe 2019	To measure the professional values of registered nurses and determine whether these values are significantly related to ethics education and years of experience.
Knecht *et al*. 2020	To gain an understanding of nursing students' attitudes and beliefs about professional values

	at entry and exit of an online registered nurse–to–bachelor of science in a nursing program that includes standalone ethics course and integrates the Code throughout the curriculum.
McNeill *et al.* 2020	To investigate nurses' perceived duty to care during disaster events
Qualitative publications (n=11)	
Murray 2003 *(Dissertation)*	To explore patients' rights as perceived by adult patients.
Kern 2005 *(Dissertation)*	To examine the development of personal nursing philosophies with nurses who left the profession.
Kalb & O'Conner-Von 2007	To ask nursing students to describe their baseline ethics-related knowledge including the concept of respect for human dignity (Provision 1), and how they practice with respect of all individuals.
Reyes-Villacomeza 2009 *(Dissertation)*	To generate a theory that explains the development of foreign-educated physicians' nursing identity.
Burnell 2011 *(Dissertation)*	To portray the attributes of compassionate care listening to the voice of patients
Clymin *et al.* 2012	To look at nurses' knowledge and beliefs with regard to the DWDA (Washington State Death with Dignity Act)
Sullivan 2012 *(Dissertation)*	To discover, describe, and analyze the culture care beliefs, values, and practices of new baccalaureate prepared registered nurses regarding the primacy of an ethical commitment to the patient
Esposito & Sollazzo 2018	To discuss various legal and ethical challenges in short-term medical missions in natural disasters
Wentworth *et al.* 2020	To evaluate students' respond to clinical nurses' breaches of standards of practice
Dellasega & Kanaskie 2021	To explore the Code's relevance during the Covid-19 pandemic
Lancaster *et al.* 2022	To analyze and describe nurses' TikTok videos featuring dancing nurses during Covid-19 pandemic

Appendix 2. The content of scientific (n=71) and non-scientific publications (n=785).

**Content**	**Scientific (n=71)**		**Non-scientific n=785)**	
	**n**	**%**	**n**	**%**
**Professional ethics and good care**	**34**	**48**	**316**	**40**
**Codes of ethics in nursing**	**5**		**98**	
ANA Code	*5*		*77*	
Codes in nursing (general)	-		*21*	
**Professional ethics**	**15**	**21**	**116**	**15**
Professional values	*10*		*8*	
Ethical dilemmas and decision making	*2*		*12*	
Morality and virtues	*2*		*15*	
Nurse's ethical behavior and boundaries	-		*11*	
Professional standards of practice	-		*6*	
Nurse's role and duties	*1*		*26*	
Professionalism and civility	-		*33*	
Collegiality	-		*5*	
**Ethical principles in patient care**	**14**	**20**	**102**	**13**
Respect	*5*		*4*	
Patient dignity			*9*	
Social justice	*4*		*4*	
Advocacy, accountability	*2*		*45*	
Patient vulnerability	*2*		-	
Social ethics/policy	-		-	
Patient's rights	*1*		-	
Commitment to care	-		-	
Compassionate care	-		-	
Ethics in nursing in general	-		*30*	
Privacy, confidentiality	-		*5*	

		Advocacy, immigrants	-		*3*	
		Patient integrity	-		*2*	
	**Ethics and law**		**2**	**3**	**10**	**1**
**Nursing practice**			**17**	**24**	**212**	**27**
	**Nursing fields**		**4**	**6**	**47**	**6**
		Perioperative nursing	1		*13*	
		Pediatric nursing	1		*2*	
		Psychiatric nursing			*2*	
		Obstetric nursing	-		*-*	
		ICU care	1		*-*	
		Public health nursing	1		*-*	
		Nursing in general	-		*17*	
		Military and prison nursing	-		*4*	
		Nephrology	-		*2*	
		Oncology	-		*2*	
		Other	-		*5*	
	**Topics in nursing**		**13**	**18**	**111**	**14**
		Disasters and pandemics	3		*21*	
		Assisted suicide	2		*7*	
		Genomics and bioethics	2		*3*	
		Racism	1		*18*	
		Cultures and religions	1		*9*	
		End-of-life care	2		*8*	
		Violence in homes and society	1		*7*	
		Cross-linguistic nursing	1		*-*	
		Quality of care and patient safety	-		*19*	
		Sexuality and health, gender	-		*9*	
		Environmental health, sustainability and climate change	-		*7*	
		Family nursing	-		*3*	
	**Clinical issues**		**1**	**1**	**44**	**6**
		Psychosocial outcomes	1		*-*	
		Medications	-		*14*	
		Immunization and infections	-		*10*	
		Pain	-		*7*	
		Care planning and documentation	-		*6*	
		Organ donation	-		*3*	
		Nutritional therapy	-		*2*	
		Use of restraints	-		*2*	
**Work environment and nurses' working life**			**13**	**18**	**133**	**17**
	**Working life**		**2**	**3**	**56**	**7**
		Workplace violence and bullying	*1*		*17*	
		Nurse's wellbeing (self-care)	*1*		*12*	
		Nurse's wellbeing (workload, fatigue and resiliency)	-		*15*	
		Health and safety	-		*9*	
		Contract and working time	-		*3*	
	**Working community and collaboration**		**7**	**10**	**31**	**4**
		Social networking in web, nurses in social media	*7*		*10*	
		Collaboration in nursing community	-		*17*	
		Safety culture	-		*4*	
	**Nurse's role**		**5**	**7**	**17**	**2**
		Nurse identity	2		*-*	
		Nurse's dual role	1		*-*	
		Nurse professional boundaries			*-*	
		Informed consent	2		*-*	
		Influence and policy-making and political advocacy	-		*12*	

Organizational activities	-		*5*	
**Nurse's rights**	**-**	**-**	**13**	**2**
**Workforce issues**	**-**	**-**	**16**	**2**
Staffing and shortage	-		*12*	
Foreign nurses	-		*2*	
Nurse's gender	-		*2*	
**Association activities and membership**	**-**	**-**	**70**	**9**
**Leadership**	**-**		**23**	**3**
Nursing leadership in general	-		*16*	
Health care administration and policy	-		*7*	
**Education**	**6**	**8**	**17**	**2**
Teaching, mentoring and instructing	4		13	
Student's ethical behavior	2		4	
**Research**	**1**	**1**	**3**	**-**
Evidence based practice			*2*	
Nurse conducted research	1		-	
Research ethics	-		*1*	
**Other**	**-**	**-**	**11**	**1**
